# Charge Transport and Thermoelectric Performance of Bornite Controlled by Cu Non-Stoichiometry

**DOI:** 10.3390/molecules31152641

**Published:** 2026-07-29

**Authors:** Hyungil Kim, Hyemin Oh, Il-Ho Kim

**Affiliations:** Department of Materials Science and Engineering, College of Engineering, Korea National University of Transportation, Chungju 27469, Republic of Korea; 100years-kim@naver.com (H.K.); ohm050510@naver.com (H.O.)

**Keywords:** thermoelectric, Cu_5_FeS_4_, bornite, off-stoichiometric, non-stoichiometric

## Abstract

Cu-deficient bornite Cu_5−x_FeS_4_ (x = 0–0.20) samples were synthesized by mechanical alloying (MA) followed by hot pressing (HP) to investigate the effects of Cu-site non-stoichiometry on structural evolution, charge transport, and thermoelectric performance. X-ray diffraction confirmed single-phase bornite formation after MA, whereas a minor chalcopyrite CuFeS_2_ secondary phase appeared in highly Cu-deficient samples after HP, indicating reduced phase stability during thermal consolidation. Rietveld refinement revealed anisotropic lattice distortion and a gradual decrease in unit-cell volume with increasing Cu deficiency. Hall-effect measurements showed that Cu vacancies act as acceptor defects, increasing the hole concentration to the order of 10^18^–10^19^ cm^−3^ while reducing carrier mobility through enhanced defect scattering. Consequently, the electrical conductivity increased, whereas the Seebeck coefficient decreased with increasing Cu deficiency. The power factor was enhanced, reaching 0.46 mW m^−1^ K^−2^ at 723 K for Cu_4.80_FeS_4_. The thermal conductivity remained low at 0.48–0.77 W m^−1^ K^−1^ owing to dominant lattice contributions and intensified phonon scattering. As a result, Cu_4.80_FeS_4_ exhibited a 52% higher ZT at 523 K than stoichiometric Cu_5_FeS_4_, demonstrating that Cu deficiency is an effective strategy for tuning carrier concentration and defect structure in bornite.

## 1. Introduction

Thermoelectric materials can directly convert waste heat into electrical energy or enable solid-state cooling, making them important for energy-harvesting and thermal-management applications [1]. Their performance is evaluated using the dimensionless figure of merit (ZT), which is governed by the coupled relationship among the Seebeck coefficient, electrical conductivity, and thermal conductivity [2]. Because these transport parameters are strongly coupled, independently optimizing them remains a fundamental challenge in thermoelectric materials research. Accordingly, considerable efforts have been devoted to materials-design strategies that decouple charge and heat transport. Chalcogenide compounds have attracted attention as promising thermoelectric materials owing to their complex crystal structures and intrinsically low lattice thermal conductivity [3], and these favorable characteristics are also commonly observed in Cu-based chalcogenides [4]. In these materials, non-stoichiometry, particularly Cu deficiency, can generate Cu vacancies that act as acceptor defects and increase the hole concentration. This carrier modulation typically enhances electrical conductivity while reducing the Seebeck coefficient, representing a characteristic charge-transport behavior of Cu-based chalcogenides. Similar trends have been reported in permingeatite Cu_3_SbSe_4_ [5,6,7,8] and famatinite Cu_3_SbS_4_ [9,10]. Therefore, controlling Cu non-stoichiometry, either through Cu excess or Cu deficiency, provides an effective strategy for simultaneously tuning carrier concentration and structural disorder, thereby modifying both electrical and thermal transport properties.

Bornite Cu_5_FeS_4_ is a representative Cu-based chalcogenide with a complex crystal structure and intrinsically low lattice thermal conductivity. Its low-temperature orthorhombic structure is characterized by partial and split occupancy of Cu sites, whereas Fe occupies relatively ordered sites, resulting in a highly disordered and structurally flexible Cu sublattice [11]. Bornite is also a polymorphic sulfide that undergoes two successive temperature-induced structural phase transitions, from the low-temperature orthorhombic structure to an intermediate cubic structure and subsequently to a high-temperature cubic structure [12,13]. The partial Cu-site occupancy and associated cation disorder enhance phonon scattering and contribute to the intrinsically low lattice thermal conductivity of bornite [11,14]. Moreover, cation ordering and vacancy redistribution can modify carrier concentration and phonon transport, indicating that the defect structure plays a central role in determining its coupled electrical and thermal transport properties [12,13,14,15]. Despite these favorable structural characteristics, reproducible bulk synthesis and precise control of carrier transport remain important requirements for improving the thermoelectric performance of bornite-based materials.

Previous studies have explored synthesis optimization, nanostructuring, non-stoichiometry, and elemental substitution as strategies for modifying the thermoelectric properties of bornite. Mechanical alloying based on high-energy ball milling promotes bornite phase formation while inducing nanostructuring; the resulting grain refinement and defect generation enhance phonon scattering and reduce thermal conductivity [16,17]. Colloidal synthesis has enabled the preparation of non-stoichiometric bornite nanostructures, while core–shell architectures and twin boundaries have been investigated as microstructural approaches to improving thermoelectric performance [18,19,20]. In addition, Co and Ni doping and Se substitution have been used to modify carrier concentration, crystal structure, and microstructure [17,21,22,23]. Of particular relevance to the present work, Long et al. [15] systematically investigated Cu-deficient and Cu-rich bornite-related compositions and demonstrated that thermoelectric performance can be optimized through control of the nominal Cu^+^ content and cation-vacancy concentration. Their study established the broad compositional dependence of the thermoelectric properties of bornite; however, carrier concentration and mobility were not directly measured, and the increase in hole concentration associated with Cu deficiency was interpreted primarily using formal oxidation-state considerations and electrical transport behavior.

In our previous studies, dense and homogeneous bulk bornite samples were fabricated by mechanical alloying (MA) and hot pressing (HP) [24], while elemental doping effectively controlled the carrier concentration and electrical conductivity [25]. Fe-site non-stoichiometry in Cu_5_Fe_1±y_S_4_ was also shown to induce defects and lattice distortion, thereby reducing thermal conductivity and modifying charge transport [26]. These findings demonstrate the effectiveness of doping and non-stoichiometry for defect-mediated optimization of thermoelectric properties. However, previous bornite studies have focused mainly on Fe-site compositional modification, leaving the effects of Cu-site non-stoichiometry comparatively unexplored. This study therefore investigates progressive Cu deficiency at fixed Fe content under MA–HP processing. Phase evolution, Cu-deficiency-induced phase instability, anisotropic lattice distortion, and microstructure are correlated with directly measured hole concentration and carrier mobility to clarify their effects on the electrical, thermal, and thermoelectric properties of bornite.

## 2. Results and Discussion

**Figure 1:**
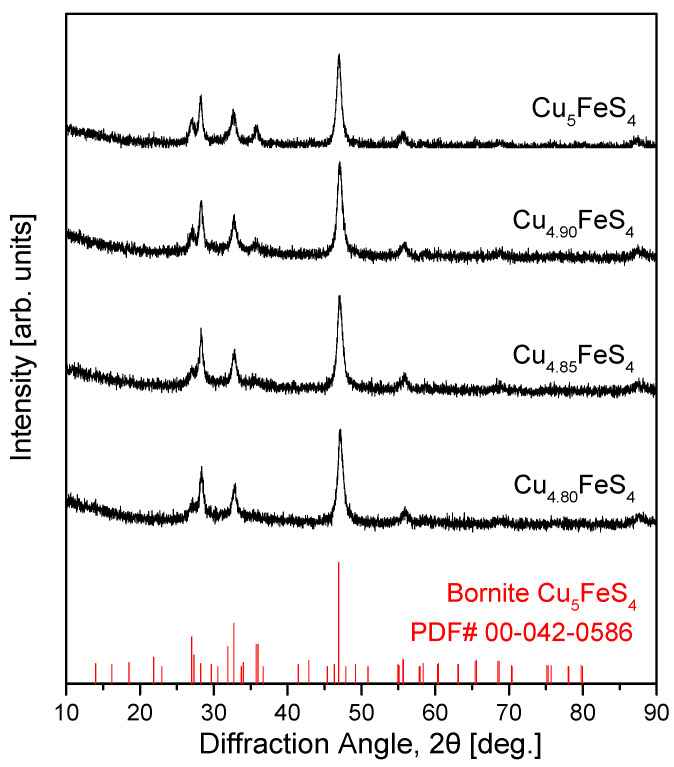
XRD patterns of mechanically alloyed Cu_5−x_FeS_4_ powders. All samples exhibit diffraction peaks corresponding to the bornite phase, indicating successful phase formation without detectable secondary phases. For comparison, data for the stoichiometric Cu_5_FeS_4_ are taken from Ref. [24].

Figure 1 shows the XRD patterns of the mechanically alloyed Cu_5−x_FeS_4_ powders. The main diffraction peaks observed for all specimens are in good agreement with the standard diffraction pattern of bornite (PDF# 00-042-0586), indicating that the desired bornite phase was successfully formed by the MA process alone. Notably, no discernible secondary-phase or impurity peaks were detected despite the introduction of Cu deficiency, suggesting that Cu vacancies were accommodated within the bornite lattice over the investigated compositional range without inducing detectable phase separation. This result is consistent with previous reports that bornite has relatively high defect tolerance owing to its characteristic crystal structure, which consists of a comparatively ordered Fe sublattice and partially occupied or split-occupied Cu sites [11]. Meanwhile, the broad diffraction peaks observed for all specimens are attributed to the small crystallite size, high defect density, and residual microstrain introduced during MA, indicating that the as-milled powders possess a defect-rich nanocrystalline structure. Considering the non-equilibrium nature of MA, the formation of the bornite phase is likely stabilized by defect-assisted structural accommodation rather than by thermodynamic equilibrium. These structural features are expected to influence not only the subsequent crystallite growth and defect relaxation during sintering but also phonon-scattering behavior. Therefore, Cu deficiency in the MA powders is considered to be primarily incorporated as lattice defects and microstructural imperfections within the bornite structure, rather than promoting phase separation at this stage.

**Figure 2:**
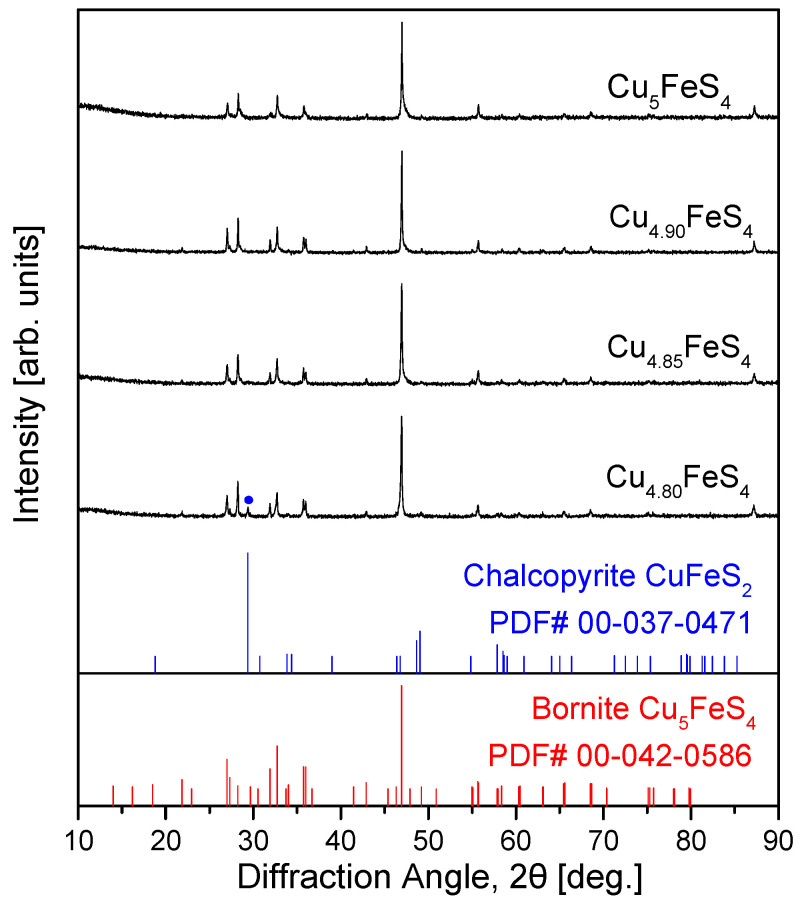
XRD patterns of hot-pressed Cu_5−x_FeS_4_ bulk samples. The bornite phase is predominantly retained, while a minor chalcopyrite CuFeS_2_ secondary phase emerges in highly Cu-deficient compositions. For comparison, data for the stoichiometric Cu_5_FeS_4_ are taken from Ref. [24].

Figure 2 presents the XRD patterns of the sintered Cu_5−x_FeS_4_ bulk specimens prepared by HP, revealing the structural evolution after sintering. Although the bornite phase is predominantly retained in all non-stoichiometric samples, additional diffraction peaks corresponding to chalcopyrite CuFeS_2_ (PDF# 00-037-0471) are observed in the Cu_4.80_FeS_4_ sample, indicating the formation of a minor secondary phase at higher Cu deficiency. This behavior can be attributed to the thermally activated redistribution of Cu vacancies during the HP process. While Cu deficiency can be accommodated within the bornite lattice under the non-equilibrium conditions of MA, thermal activation during sintering likely promotes atomic redistribution, causing the single-phase stability of bornite to be exceeded beyond a critical Cu-deficiency level and leading to secondary-phase formation. Because the charge-transport behavior and structural disorder of bornite are highly sensitive to Cu-site occupancy and vacancy concentration [14,15], the observed secondary-phase formation is considered to be closely related to the compositional dependence of phase stability. The chalcopyrite phase is likely formed through local compositional imbalance under Cu-deficient conditions, where relatively Fe-rich regions become stabilized as CuFeS_2_. Meanwhile, the overall narrowing of the diffraction peaks after sintering indicates crystallite growth and partial defect relaxation; however, this effect is less pronounced in highly Cu-deficient samples, in which broadened diffraction features remain for several reflections. This residual peak broadening is attributed to the combined effects of lattice distortion, compositional inhomogeneity, and minor secondary-phase formation induced by increased Cu deficiency. Compared with the stoichiometric sample reported in our previous study [24], which retained a stable single-phase bornite structure after HP, the present results indicate that phase stability gradually decreases with increasing Cu deficiency. This trend is consistent with previous reports on Cu-based chalcogenides, such as permingeatite [6], in which single-phase stability becomes difficult to maintain when the degree of non-stoichiometry exceeds a certain limit.

The Rietveld refinement results summarized in Table 1 indicate that the orthorhombic bornite structure is generally retained in the Cu-deficient samples, while the lattice constants and microstructural parameters vary with Cu deficiency. Compared with stoichiometric Cu_5_FeS_4_ [24], the Cu-deficient samples exhibit anisotropic lattice distortion, characterized by increases in the *a*- and *c*-axis lattice constants and a decrease in the *b*-axis constant, resulting in an overall unit-cell volume contraction of 0.44–0.65%. This behavior suggests that the introduction of Cu vacancies does not simply induce isotropic lattice contraction, but instead promotes crystallographic-direction-dependent structural rearrangement accompanied by local lattice distortion. Although Cu deficiency generally tends to reduce the lattice volume in various Cu-based chalcogenides, the detailed lattice response strongly depends on the crystal structure. For example, decreases in both the *a*- and *c*-axis lattice parameters, together with reduced tetragonality, have been reported for tetragonal famatinite [10]. These comparisons indicate that Cu deficiency commonly affects the structural parameters of Cu-based chalcogenides, although the specific anisotropic response is structure-dependent. In addition, the average crystallite size initially increases relative to that of the stoichiometric sample but decreases again with further increasing Cu deficiency. This trend can be interpreted in terms of competing effects: at low Cu-deficiency levels, Cu vacancies may enhance atomic diffusion and promote crystallite growth during sintering, whereas higher vacancy concentrations increase lattice distortion and defect accumulation, thereby restricting crystallite growth and grain-boundary migration.

**Figure 3:**
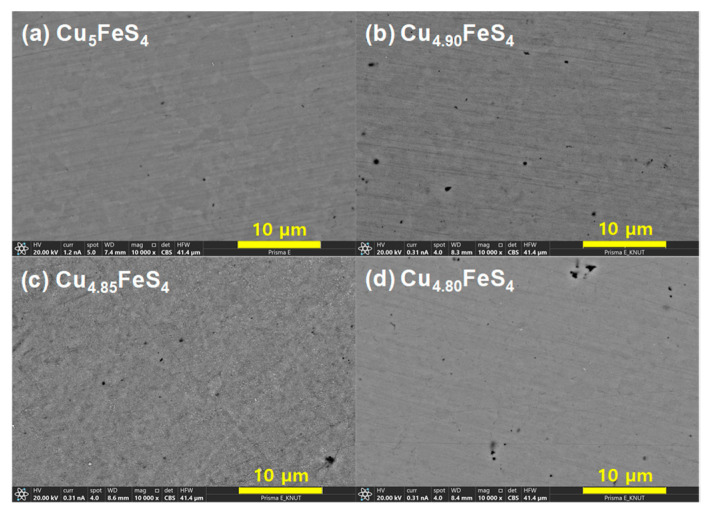
BSE−SEM images of hot-pressed Cu_5−x_FeS_4_ samples: (**a**) Cu_5_FeS_4_, (**b**) Cu_4.90_FeS_4_, (**c**) Cu_4.85_FeS_4_, and (**d**) Cu_4.80_FeS_4_. All samples exhibit dense microstructures with high relative density and negligible porosity. For comparison, data for the stoichiometric Cu_5_FeS_4_ are taken from Ref. [24].

Figure 3 shows cross-sectional SEM micrographs of the hot-pressed Cu_5−x_FeS_4_ specimens obtained in backscattered electron (BSE) mode. All samples exhibit highly dense microstructures without noticeable coarse pores, microcracks, or distinct phase-separated regions within the observed magnification range, indicating that the MA-derived powders were effectively consolidated during HP. This observation is consistent with the high relative densities of 98.2–99.8% listed in Table 1. The nearly uniform BSE contrast further suggests the absence of pronounced compositional inhomogeneity or secondary phases with a high volume fraction on the micrometer scale. The overall morphology and densification behavior remain essentially unchanged with increasing Cu deficiency, indicating that Cu deficiency has only a limited effect on microstructural evolution within the investigated compositional range. Although small dark-contrast regions are observed in some samples, these features are very limited in both size and fraction and may be associated with local compositional fluctuations, micropores, or trace secondary phases. Nevertheless, continuous phase separation or distinct grain-boundary precipitates are not observed. Therefore, the bulk specimens prepared in this study are considered to maintain dense microstructures predominantly composed of the bornite matrix.

**Figure 4:**
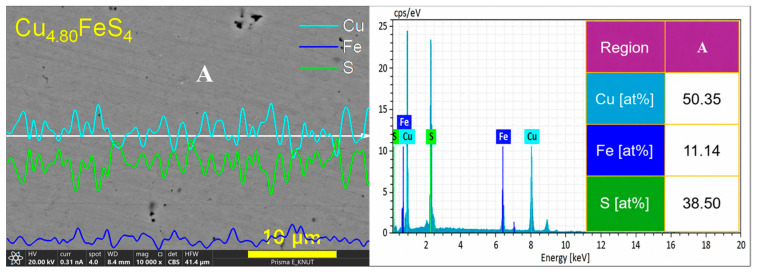
EDS analysis of Cu_5−x_FeS_4_, including line scan and point analysis. A relatively homogeneous elemental distribution is observed without significant compositional segregation. For comparison, data for the stoichiometric Cu_5_FeS_4_ are taken from Ref. [24].

Figure 4 presents the EDS line-scan and point-analysis results for the Cu_4.80_FeS_4_ specimen. The Cu, Fe, and S signals remain nearly uniform across the analyzed region, with no clear evidence of localized elemental enrichment or depletion, and the point analysis for Region A confirms that the average composition agrees well with the nominal Cu_4.80_FeS_4_ composition. These results indicate that the matrix phase of the hot-pressed specimen is predominantly composed of compositionally homogeneous bornite, consistent with the uniform BSE–SEM contrast shown in Figure 3. Although the XRD results in Figure 2 reveal a small amount of chalcopyrite CuFeS_2_ in the Cu_4.80_FeS_4_ specimen, this secondary phase is not clearly resolved by EDS analysis. This discrepancy is likely due to the very low volume fraction and fine spatial distribution of the chalcopyrite phase; XRD probes the long-range crystallographic order of the bulk specimen and can detect minor crystalline phases, whereas EDS analyzes a limited local region and may not capture such phases unless they are included within the selected line-scan path or point-analysis area. If the chalcopyrite phase is dispersed as submicrometer-scale particles, its signal may also be averaged with that of the surrounding bornite matrix. Moreover, because chalcopyrite CuFeS_2_ and bornite Cu_5_FeS_4_ belong to the same Cu–Fe–S system and contain the same constituent elements, their small compositional and average atomic-number contrast makes them difficult to distinguish by BSE contrast or conventional EDS analysis. Therefore, the chalcopyrite phase detected by XRD in the Cu_4.80_FeS_4_ specimen is considered to be present as a very fine, low-volume-fraction secondary phase below the practical spatial resolution and detection sensitivity of the local EDS measurements used here.

**Figure 5:**
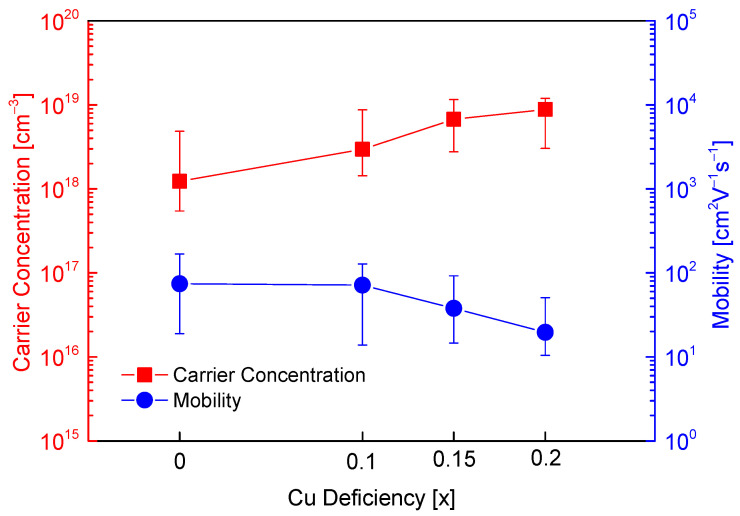
Carrier concentration and mobility of Cu_5−x_FeS_4_ samples measured at room temperature. Increasing Cu deficiency leads to an increase in carrier concentration and a decrease in mobility due to enhanced defect scattering. For comparison, data for the stoichiometric Cu_5_FeS_4_ are taken from Ref. [24].

Figure 5 presents the Cu-composition dependence of the carrier concentration and mobility of the Cu_5−x_FeS_4_ samples. With increasing Cu deficiency, the carrier (hole) concentration gradually increases from 1.2 × 10^18^ to 8.8 × 10^18^ cm^−3^, which is consistent with the formation of negatively charged Cu vacancies ($V_{Cu}^{-}$) as acceptor-type defects and the consequent generation of holes [15]. The preferential accommodation of the deficiency at Cu sites can be understood from the structural and electronic characteristics of bornite. In orthorhombic bornite, Fe occupies relatively regular and nearly fully occupied FeS_4_ tetrahedral sites, whereas Cu is distributed over multiple partially occupied split sites with threefold or distorted fourfold coordination, making the Cu sublattice structurally more flexible for accommodating cation deficiency [11]. In a formal ionic description, stoichiometric Cu_5_FeS_4_ can be represented predominantly as ${Cu}_{5}^{+}{Fe}^{3+}S_{4}^{2-}$; thus, the removal of Cu^+^ can be charge-compensated by the oxidation of a fraction of Cu^+^ to Cu^2+^, or equivalently by hole generation [15]. Spectroscopic studies of non-stoichiometric bornite have shown that small compositional variations significantly modify the Cu–S electronic structure and sulfur-ligand hole population [27]. Therefore, hole generation associated with Cu deficiency may alter the occupation of Cu–S-derived electronic states, although the specific depopulation of Cu 3d–S 3p antibonding states and the resulting electronic stabilization were not directly verified in the present study.

In contrast, the carrier mobility decreases from 74.4 to 19.6 cm^2^ V^−1^ s^−1^ with increasing Cu deficiency, indicating that the increased concentration of Cu vacancies and associated off-stoichiometric defects enhances carrier scattering. In addition, as shown in Table 1, the Cu-deficient samples exhibit anisotropic changes in the lattice parameters together with an overall reduction in unit-cell volume. These structural modifications are expected to increase the defect density and induce local lattice distortion, further intensifying carrier scattering and contributing to the mobility reduction. These trends are consistent with previous reports on Cu-deficient Cu-based chalcogenides. For example, in permingeatite, Cu vacancies have been reported to act as acceptor defects, increasing carrier concentration while simultaneously reducing carrier mobility [5,6]. Similar defect-induced modifications of charge-transport properties caused by Cu non-stoichiometry have also been reported for famatinite [10].

**Figure 6:**
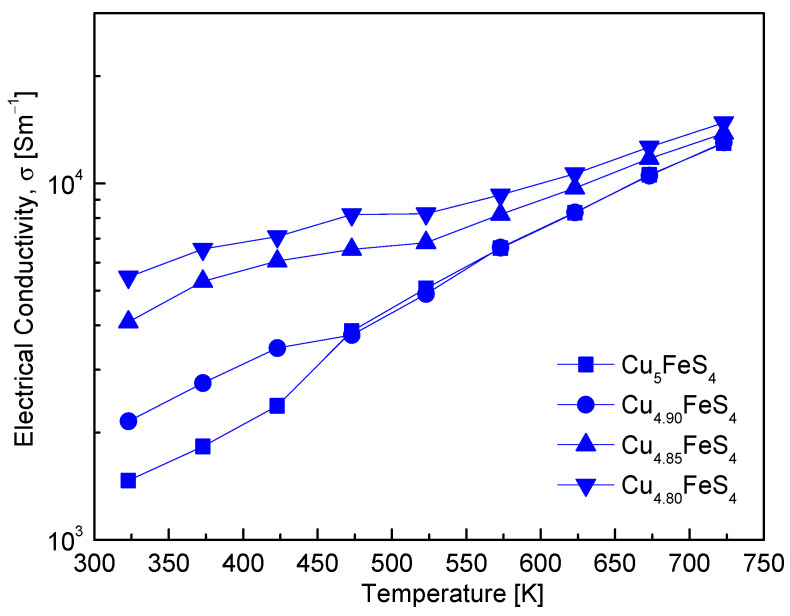
Temperature dependence of electrical conductivity of Cu_5−x_FeS_4_ samples. Electrical conductivity increases with both increasing temperature and Cu deficiency. For comparison, data for the stoichiometric Cu_5_FeS_4_ are taken from Ref. [24].

Figure 6 shows the temperature dependence of the electrical conductivity (σ) of the Cu_5−x_FeS_4_ samples as a function of Cu deficiency. All samples exhibit increasing σ with increasing temperature over the measured range of 323–723 K, indicating typical semiconducting transport behavior. For stoichiometric Cu_5_FeS_4_, σ increases from 1.5 × 10^3^ S m^−1^ at 323 K to 1.3 × 10^4^ S m^−1^ at 723 K, corresponding to an approximately one-order-of-magnitude increase. The Cu-deficient samples exhibit higher electrical conductivity than the stoichiometric sample over the entire temperature range. At 323 K, σ increases from 2.2 × 10^3^ to 5.5 × 10^3^ S m^−1^ with increasing Cu deficiency, which is consistent with the increase in carrier concentration shown in Figure 5. Although carrier mobility decreases with Cu deficiency, the increase in carrier concentration dominates the electrical transport, resulting in enhanced σ. In terms of temperature dependence, stoichiometric Cu_5_FeS_4_ and Cu_4.90_FeS_4_ show relatively steep increases in σ at lower temperatures, whereas Cu_4.85_FeS_4_ and Cu_4.80_FeS_4_ exhibit more gradual increases because their carrier concentrations are already high at low temperatures, reducing the relative contribution of thermally activated carrier generation. At a given temperature, σ increases monotonically with Cu deficiency in the low-temperature region, whereas the difference among the samples becomes smaller at elevated temperatures. In particular, σ converges to approximately (1.3–1.5) × 10^4^ S m^−1^ at 723 K, suggesting that the compositional dependence becomes less pronounced as thermally excited carriers increasingly contribute to charge transport. It should be noted that the x = 0.20 sample contains a minor chalcopyrite phase, as confirmed by XRD; therefore, although the enhanced electrical conductivity is mainly attributed to the carrier-doping effect induced by Cu deficiency, a possible contribution from slight structural inhomogeneity cannot be completely excluded. Overall, these results demonstrate that Cu deficiency effectively enhances the electrical conductivity of Cu_5−x_FeS_4_ over the entire temperature range, with the effect being particularly pronounced at lower temperatures.

**Figure 7:**
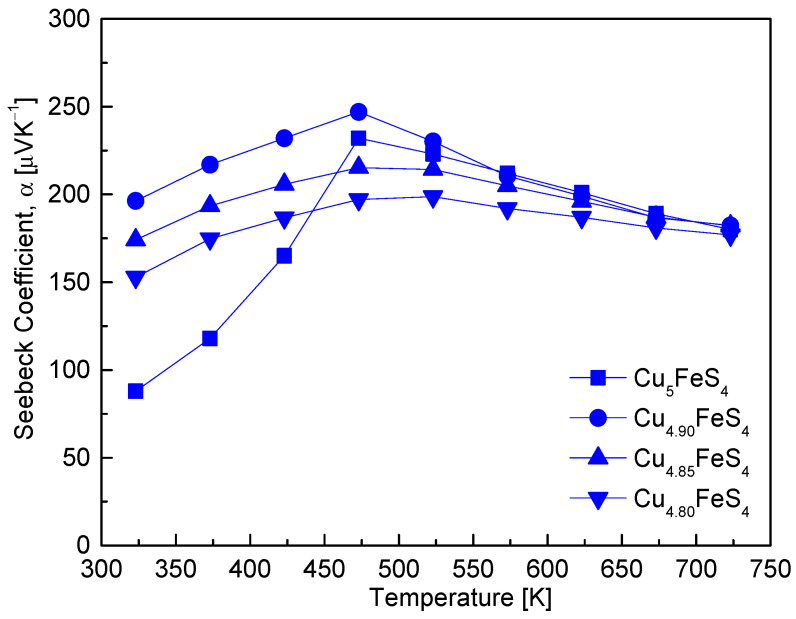
Temperature dependence of the Seebeck coefficient of Cu_5−x_FeS_4_ samples. All samples exhibit positive values, confirming p-type conduction, and the Seebeck coefficient decreases with increasing Cu deficiency. For comparison, data for the stoichiometric Cu_5_FeS_4_ are taken from Ref. [24].

Figure 7 presents the temperature dependence of the Seebeck coefficient (α) of the Cu_5−x_FeS_4_ samples as a function of Cu deficiency. All samples exhibit positive α values over the entire measured temperature range of 323–723 K, confirming p-type conduction. For stoichiometric Cu_5_FeS_4_, α increases from 88 μV K^−1^ at 323 K to a maximum of 232 μV K^−1^ at 473 K and then gradually decreases to 180 μV K^−1^ at 723 K; similar temperature-dependent behavior is observed for all samples. Bornite Cu_5_FeS_4_ undergoes two successive structural transitions upon heating: T_1_ transition from the low-temperature orthorhombic phase to the intermediate cubic phase at approximately 450–470 K, followed by T_2_ transition from the intermediate cubic phase to the high-temperature cubic phase near 538 K [12,13,14,20,23,28]. The increase in α followed by a maximum at 473 K is predominantly associated with T_1_, during which the redistribution and occupancy changes of Cu ions can affect the carrier concentration and transport behavior [22]. By contrast, α decreases smoothly between 523 and 573 K, and no separate anomaly attributable to T_2_ is resolved. A similarly weak response was observed for stoichiometric Cu_5_FeS_4_ in our previous study [24], indicating that T_2_ has only a limited effect on charge transport under the present measurement conditions. Compared with stoichiometric Cu_5_FeS_4_, the Cu-deficient samples generally show lower α values over most of the measured temperature range. However, at 323 K, Cu_4.90_FeS_4_, Cu_4.85_FeS_4_, and Cu_4.80_FeS_4_ exhibit α values of 196, 174, and 153 μV K^−1^, respectively, which are higher than that of stoichiometric Cu_5_FeS_4_. This low-temperature behavior indicates that α is governed not only by carrier concentration but also by effective mass, carrier-scattering mechanisms, and the Cu/Fe/vacancy ordering state in the low-temperature ordered phase. Therefore, although the non-stoichiometric samples have higher carrier concentrations, their larger low-temperature α values cannot be explained solely by carrier-concentration effects, implying that Cu deficiency also modifies the electronic structure and local crystal structure. Nevertheless, among the Cu-deficient samples, α decreases systematically with increasing Cu deficiency, consistent with the α ∝ m*Tn^−2/3^ relationship predicted by the single parabolic band (SPB) model [1]. This trend reflects the general behavior that an increase in hole concentration caused by acceptor-type Cu vacancies leads to a decrease in the Seebeck coefficient. At temperatures above approximately 673 K, the α values of all samples converge within a narrow range of 177–189 μV K^−1^, suggesting that the compositional dependence becomes less pronounced as thermally excited carriers increasingly contribute to charge transport.

**Figure 8:**
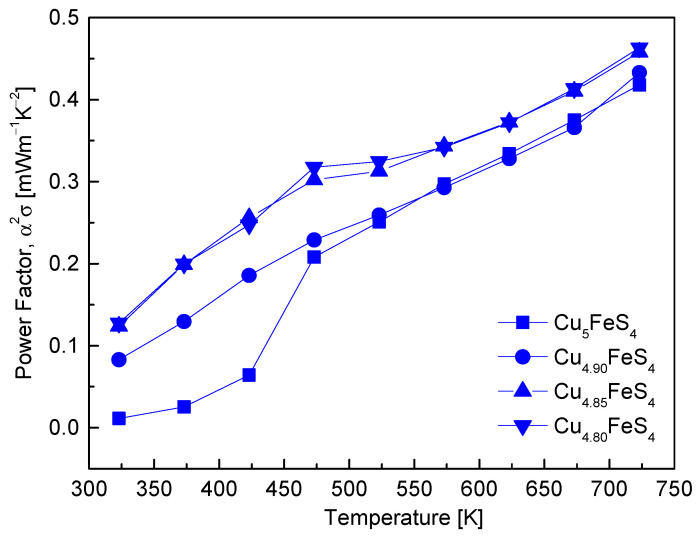
Temperature dependence of the power factor of Cu_5−x_FeS_4_ samples. Enhanced power factor values are observed in Cu-deficient compositions over the entire temperature range. For comparison, data for the stoichiometric Cu_5_FeS_4_ are taken from Ref. [24].

Figure 8 shows the temperature dependence of the power factor (PF) of the Cu_5−x_FeS_4_ samples as a function of Cu deficiency. All samples exhibit an overall increase in PF with increasing temperature, mainly due to the dominant contribution of the increasing electrical conductivity. Although the Seebeck coefficient reaches a maximum at 473 K and then gradually decreases at higher temperatures, the increase in electrical conductivity compensates for and outweighs this reduction, resulting in a continuous enhancement of PF with temperature. Compared with stoichiometric Cu_5_FeS_4_, the Cu-deficient samples show higher PF values over the entire measured temperature range. In particular, at 323 K, the PF of stoichiometric Cu_5_FeS_4_ is only approximately 0.01 mW m^−1^ K^−2^, whereas the Cu-deficient samples exhibit markedly improved values of 0.08–0.13 mW m^−1^ K^−2^. This enhancement is primarily attributed to the increased carrier concentration induced by Cu vacancies, which leads to improved electrical conductivity. Among the Cu-deficient compositions, Cu_4.85_FeS_4_ and Cu_4.80_FeS_4_ maintain relatively high PF values throughout the measured temperature range, and Cu_4.80_FeS_4_ achieves the maximum PF of 0.46 mW m^−1^ K^−2^ at 723 K. These results demonstrate that carrier-concentration tuning through Cu deficiency is an effective approach for improving the electrical transport properties of bornite, particularly in the intermediate- and low-temperature regions.

**Figure 9:**
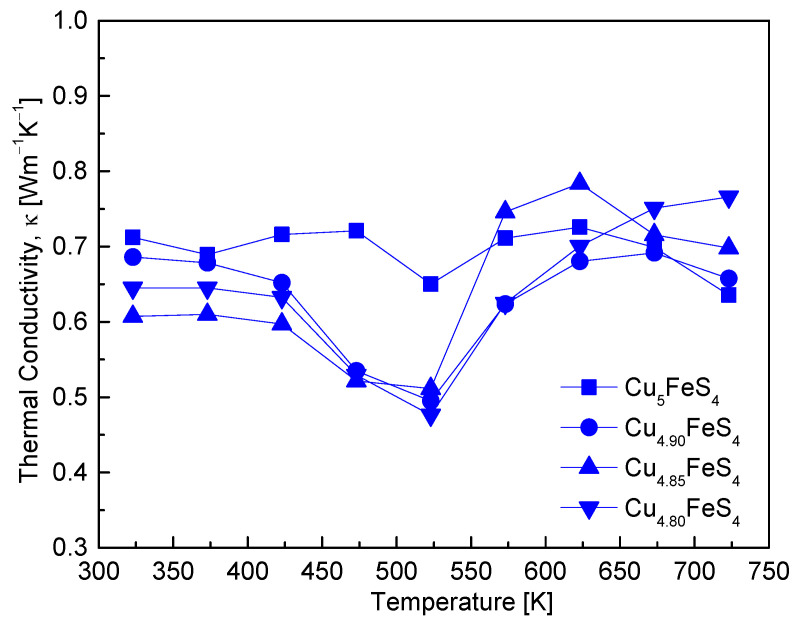
Temperature dependence of total thermal conductivity of Cu_5−x_FeS_4_ samples. Low thermal conductivity is maintained across the entire temperature range. For comparison, data for the stoichiometric Cu_5_FeS_4_ are taken from Ref. [24].

Figure 9 presents the temperature dependence of the thermal conductivity (κ) of the Cu_5−x_FeS_4_ samples as a function of Cu deficiency. For stoichiometric Cu_5_FeS_4_, κ gradually decreases from 0.71 W m^−1^ K^−1^ at 323 K, reaches a minimum at 523 K, and then exhibits non-monotonic behavior with further increasing temperature. The broad decrease in κ around 473–523 K is likely associated predominantly with the T_1_ phase transition of bornite, which involves Cu/Fe/vacancy rearrangement and changes in structural disorder, thereby modifying phonon-scattering behavior [12,14]. By contrast, only a weak variation in κ is observed in the vicinity of T_2_ phase transition, indicating that its influence on thermal transport is limited and difficult to distinguish from the overall temperature dependence. The Cu-deficient samples show a similar non-monotonic temperature dependence: κ decreases from 0.61–0.69 W m^−1^ K^−1^ at 323 K to minimum values of 0.48–0.51 W m^−1^ K^−1^ at 473–523 K, followed by an increase to 0.64–0.77 W m^−1^ K^−1^ in the 623–723 K range. Notably, Cu_4.80_FeS_4_ exhibits relatively higher κ values at elevated temperatures. This high-temperature increase in κ can be interpreted by considering that total thermal conductivity consists of lattice thermal conductivity (κ_L_) and electronic thermal conductivity (κ_E_). While κ_L_ generally decreases or varies only weakly with increasing temperature because of enhanced phonon–phonon scattering, κ_E_ increases as electrical conductivity increases, thereby contributing to the rise in total κ at higher temperatures. From the compositional perspective, the Cu-deficient samples exhibit κ values comparable to or slightly lower than that of stoichiometric Cu_5_FeS_4_ at low temperatures, whereas κ becomes relatively higher at elevated temperatures, particularly for Cu_4.80_FeS_4_ and Cu_4.85_FeS_4_.

**Figure 10:**
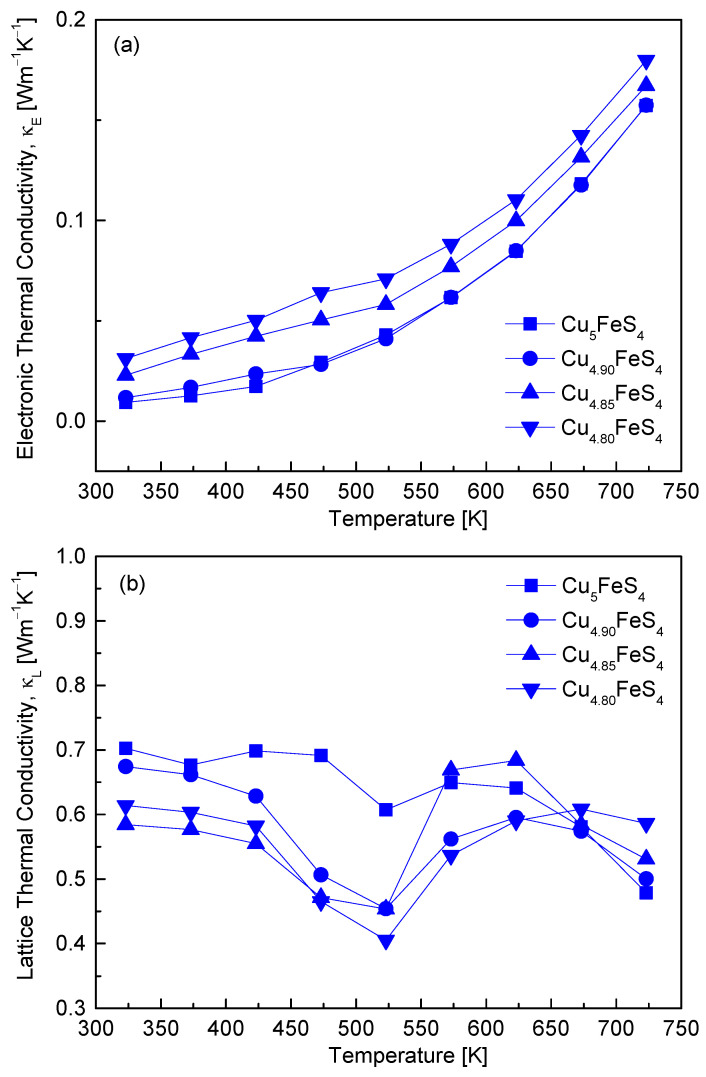
(**a**) Electronic thermal conductivity and (**b**) lattice thermal conductivity of Cu_5−x_FeS_4_ samples estimated using the Wiedemann–Franz law. The lattice contribution dominates the total thermal conductivity. For comparison, data for the stoichiometric Cu_5_FeS_4_ are taken from Ref. [24].

Figure 10a shows the temperature dependence of the electronic thermal conductivity (κ_E_) of the Cu_5−x_FeS_4_ samples as a function of Cu deficiency. For all samples, κ_E_ increases overall with increasing temperature, and the Cu-deficient samples exhibit higher κ_E_ values than the stoichiometric sample. For example, κ_E_ increases from approximately 0.01 W m^−1^ K^−1^ for Cu_5_FeS_4_ to 0.03 W m^−1^ K^−1^ for Cu_4.80_FeS_4_ at 323 K, and from 0.16 W m^−1^ K^−1^ for Cu_5_FeS_4_ to approximately 0.18 W m^−1^ K^−1^ for Cu_4.80_FeS_4_ at 723 K. This trend is consistent with the enhanced electrical conductivity discussed above and can be explained by the Wiedemann–Franz relation, κ_E_ = LσT [1], in which increases in electrical conductivity and temperature directly contribute to higher κ_E_ values. In the Cu-deficient samples, Cu vacancies increase the hole concentration, thereby enhancing electrical conductivity and consequently increasing the electronic contribution to thermal transport. Nevertheless, κ_E_ remains a relatively small fraction of the total thermal conductivity over the entire measured temperature range, indicating that heat transport in this system is still dominated primarily by the lattice thermal conductivity.

Figure 10b presents the temperature dependence of the lattice thermal conductivity (κ_L_) of the Cu_5−x_FeS_4_ samples as a function of Cu deficiency. Compared with the total thermal conductivity shown in Figure 9, κ_L_ constitutes the dominant contribution to thermal transport. For stoichiometric Cu_5_FeS_4_, κ_L_ gradually decreases from 0.70 W m^−1^ K^−1^ at 323 K to 0.61 W m^−1^ K^−1^ at 523 K and further to 0.48 W m^−1^ K^−1^ at 723 K. The Cu-deficient samples exhibit lower κ_L_ values than the stoichiometric sample, particularly at low temperatures and near the T_1_ phase-transition region, indicating that the introduction of Cu vacancies enhances phonon scattering through increased structural disorder and point-defect scattering. In particular, Cu_4.80_FeS_4_ reaches a minimum κ_L_ of approximately 0.41 W m^−1^ K^−1^ at 523 K, which is markedly lower than that of stoichiometric Cu_5_FeS_4_, demonstrating the effectiveness of Cu deficiency in suppressing phonon transport. However, above 573 K, κ_L_ in some Cu-deficient samples increases again or becomes comparable to, or even higher than, that of the stoichiometric sample. This behavior cannot be explained solely by point-defect scattering and is likely related to temperature-dependent changes in structural disorder, including cation/vacancy rearrangement and phase-transition-associated modifications in phonon-scattering mechanisms. Overall, the bornite system maintains relatively low thermal conductivity, with the lattice contribution governing the heat transport. Cu deficiency effectively reduces κ_L_ at low temperatures and around the phase-transition region, but the concurrent increase in κ_E_ caused by enhanced electrical conductivity partially offsets the reduction in total thermal conductivity at higher temperatures.

**Figure 11:**
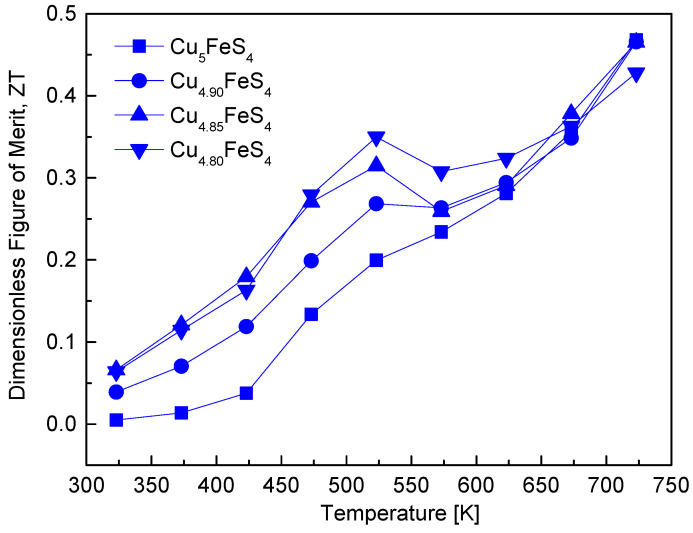
Temperature dependence of the dimensionless figure of merit (ZT) of Cu_5−x_FeS_4_ samples. Cu-deficient samples exhibit enhanced ZT values in the intermediate temperature range, while the differences diminish at higher temperatures. For comparison, data for the stoichiometric Cu_5_FeS_4_ are taken from Ref. [24].

Figure 11 shows the temperature dependence of the dimensionless thermoelectric figure of merit (ZT) of the Cu_5−x_FeS_4_ samples as a function of Cu deficiency. All samples exhibit an overall increase in ZT with increasing temperature, which is attributed to the enhanced power factor combined with the retention of low thermal conductivity at elevated temperatures. For stoichiometric Cu_5_FeS_4_, ZT increases from a very low value of 0.005 at 323 K to a maximum of 0.47 at 723 K. In contrast, the Cu-deficient samples generally show higher ZT values in the low- and intermediate-temperature regions, with the most pronounced enhancement observed at 523 K. In particular, Cu_4.80_FeS_4_ exhibits a ZT of 0.35 at 523 K, corresponding to a 52% improvement over the stoichiometric sample (ZT = 0.23) at the same temperature. However, the compositional differences gradually diminish with increasing temperature. At 623 K, the ZT values of all samples converge within a narrow range of 0.28–0.32, and at 723 K, all samples except Cu_4.80_FeS_4_ reach the same maximum ZT of 0.47, indicating that the influence of Cu deficiency becomes much less significant at higher temperatures. These results suggest that Cu deficiency is most effective in enhancing thermoelectric performance in the intermediate-temperature region, particularly around 473–573 K, whereas its beneficial effect is partially offset at higher temperatures by the increased electronic thermal conductivity associated with enhanced electrical conductivity and by the reduced rate of power-factor improvement. In addition, a temporary slowdown in the increase in ZT is observed around 573 K, which is likely related to changes in charge and heat transport associated with the T_1_ phase transition for bornite. In this temperature range, concurrent variations in the Seebeck coefficient, electrical conductivity, and thermal conductivity collectively affect the ZT behavior. These results indicate that Cu deficiency effectively increases the carrier concentration and improves the power factor, but its overall contribution to ZT is temperature-dependent. The ZT enhancement observed in the present Cu-deficient bornite system is also consistent with previous reports on other Cu-based chalcogenide thermoelectric materials. For example, Cu-deficient permingeatite Cu_2.925_SbSe_4_ exhibited a ZT of 0.50 at 673 K, representing a 2.5-fold improvement over stoichiometric Cu_3_SbSe_4_ (ZT = 0.20) [5]. These comparisons demonstrate that carrier engineering through Cu-vacancy introduction is an effective strategy across a broad range of Cu-based chalcogenides, and the present results further confirm that this approach is also effective in bornite, particularly in the intermediate-temperature region.

## 3. Materials and Methods

Non-stoichiometric Cu_5−x_FeS_4_ (x = 0.10, 0.15, and 0.20) samples were synthesized using high-purity elemental powders of Cu (99.9%, <45 μm), Fe (99.9%, <53 μm), and S (99.99%, <75 μm) (Kojundo Chemical Laboratory Co., Ltd., Saitama, Japan) as starting materials. The powders were accurately weighed according to the nominal compositions and thoroughly mixed to ensure compositional homogeneity. A 20 g batch of the mixed powder was loaded into a 500 mL hardened-steel vial together with stainless-steel balls with a diameter of 5 mm, and the ball-to-powder weight ratio (BPR) was fixed at 20:1. Mechanical alloying (MA) was performed using a planetary ball mill (Pulverisette5, Fritsch GmbH, Idar-Oberstein, Germany) operated at 350 rpm for 6 h under a high-purity (99.999%) Ar atmosphere to minimize oxidation during milling.

The mechanically alloyed powders were subsequently loaded into a graphite die and consolidated into dense bulk specimens by hot pressing (HP). Sintering was performed under vacuum using a uniaxial hot-pressing system (JM-HP20, Jeongmin Industrial Co., Ltd., Seoul, Republic of Korea). The samples were heated to 723 K and held for 2 h under an applied pressure of 70 MPa, which was maintained throughout both the heating and holding stages to promote uniform densification. After sintering, the specimens were furnace-cooled to room temperature to minimize residual stresses caused by thermal shock. The MA–HP processing conditions used in this study were selected based on our previous work, in which single-phase stoichiometric bornite with a high relative density was successfully obtained [24].

Phase identification and crystal-structure analysis were performed by X-ray diffraction (XRD; D8 Advance, Bruker AXS GmbH, Karlsruhe, Germany) using Cu Kα radiation (λ = 0.15405 nm). Diffraction patterns were collected over a 2θ range of 10–90° with a step size of 0.02° and a counting time of 0.4 s per step. The obtained diffraction data were analyzed by Rietveld refinement using the TOPAS software package (version 4.1, Bruker AXS GmbH, Karlsruhe, Germany) to determine the phase constitution and crystallographic parameters. After correcting for instrumental broadening using a standard reference material (NIST SRM 1976; corundum), the diffraction peaks were fitted with Lorentzian profiles to determine their full widths at half maximum (FWHMs), and the average crystallite size was subsequently estimated using the Scherrer equation. The microstructure was observed on polished surfaces using scanning electron microscopy (SEM; Prisma E, Thermo Fisher Scientific Inc., Waltham, MA, USA), and the elemental composition and spatial distribution were examined by energy-dispersive X-ray spectroscopy (EDS; Quantax200, Bruker AXS GmbH, Karlsruhe, Germany). Quantitative EDS analysis was conducted using the characteristic X-ray lines of Cu (Kα: 8.040 keV and Lα: 0.928 keV), Fe (Kα: 6.401 keV and Lα: 0.705 keV), and S (Kα: 2.309 keV).

The electrical transport properties were evaluated at room temperature using a Hall measurement system (Model 7065, Keithley Instruments LLC, Solon, OH, USA) in the van der Pauw configuration. The carrier concentration was estimated from the measured Hall coefficient (R_H_) using the single-carrier approximation, n = (eR_H_)^−1^, and the carrier mobility was calculated as μ = |R_H_|σ. The Seebeck coefficient (α) and electrical conductivity (σ) were simultaneously measured over the temperature range of 323–723 K using a thermoelectric measurement system (ZEM-3, Advance-Riko Inc., Yokohama, Japan). The thermal diffusivity (D) was measured using a laser flash analyzer (LFA417 HyperFlash, Netzsch GmbH, Selb, Germany), and the bulk density (d) was determined by the Archimedes method. The specific heat capacity (c_p_) was estimated using the Dulong–Petit approximation, and the thermal conductivity (κ) was calculated using the relation κ = dc_p_D. The power factor (PF = α^2^σ) and dimensionless thermoelectric figure of merit (ZT = α^2^σT/κ) were then calculated over the same temperature range using the measured transport parameters. All thermoelectric transport measurements were conducted parallel to the hot-pressing direction to maintain a consistent measurement geometry.

## 4. Conclusions

The effects of Cu non-stoichiometry on the structural stability, charge transport, and thermoelectric performance of Cu-deficient bornite Cu_5−x_FeS_4_ bulk samples fabricated by mechanical alloying followed by hot pressing were systematically investigated. During mechanical alloying, single-phase bornite was successfully formed; however, after hot pressing, a secondary chalcopyrite CuFeS_2_ phase appeared in the highly Cu-deficient composition, indicating that excessive Cu deficiency reduces the phase stability of bornite during thermal consolidation. With increasing Cu deficiency, anisotropic lattice distortion was observed, characterized by increases in the *a*- and *c*-axis lattice constants and a decrease in the *b*-axis constant, together with a reduction in unit-cell volume. This behavior is attributed to local structural rearrangement and lattice distortion induced by the introduction of Cu vacancies. The Cu vacancies acted as acceptor defects, increasing the carrier (hole) concentration, whereas the carrier mobility decreased owing to enhanced defect scattering. Consequently, the electrical conductivity increased while the Seebeck coefficient decreased, reflecting typical p-type semiconducting transport behavior. Despite these opposing changes, the increase in electrical conductivity dominated the electrical transport response, leading to a significant enhancement of the power factor in the Cu-deficient samples. The thermal conductivity remained low over the entire measured temperature range and was governed primarily by the lattice contribution. Cu deficiency effectively reduced the lattice thermal conductivity at low temperatures and near the phase-transition region through enhanced point-defect scattering; however, at higher temperatures, the reduction in total thermal conductivity was partially offset by the increased electronic thermal conductivity associated with enhanced electrical conductivity. As a result, Cu deficiency improved the thermoelectric performance most effectively in the intermediate-temperature region, and Cu_4.80_FeS_4_ exhibited a ZT value at 523 K more than 50% higher than that of the stoichiometric sample. In contrast, the ZT differences among the compositions became much smaller at higher temperatures, indicating that the beneficial effect of Cu deficiency is limited in the high-temperature region. These findings demonstrate that Cu non-stoichiometry is an effective strategy for simultaneously tuning carrier concentration and defect structure in the bornite system, providing useful guidance for the design and optimization of bornite-based thermoelectric materials for intermediate-temperature applications.

## Figures and Tables

**Table 1:** Crystallographic parameters, relative densities, and Rietveld refinement indices of hot-pressed Cu_5−x_FeS_4_ samples. The anisotropic evolution of lattice parameters and the corresponding reduction in unit cell volume with increasing Cu deficiency are summarized. For comparison, data for the stoichiometric Cu_5_FeS_4_ are taken from Ref. [24].

Specimen	Lattice Constant [nm]			Cell Volume [nm^3^]	Crystallite Size [nm]	Relative Density [%]	R_wp_	R_p_	GOF
	*a*	*b*	*c*				[%]	[%]	
Cu_5_FeS_4_	1.0874(4)	2.1806(6)	1.0991(3)	2.6062	64	99.8	10.36	8.17	1.44
Cu_4.90_FeS_4_	1.0915(2)	2.1553(5)	1.1030(3)	2.5948	87	98.9	8.27	6.50	1.24
Cu_4.85_FeS_4_	1.0913(1)	2.1546(5)	1.1022(2)	2.5918	76	98.8	8.18	6.44	1.17
Cu_4.80_FeS_4_	1.0912(3)	2.1538(6)	1.1016(3)	2.5892	69	98.2	8.83	6.96	1.28

## Data Availability

The original contributions presented in this study are included in the article; further inquiries can be directed to the corresponding author.
